# A Narrative Review on Magnesium Sulfate as a Game Changer in Reducing ICU Stays in Organophosphate Poisoning Cases

**DOI:** 10.7759/cureus.65481

**Published:** 2024-07-26

**Authors:** Manikanta Nelakuditi, Sunil Kumar, Suhail M Shaikh, Avinash Parepalli, M Jayanth Kumar

**Affiliations:** 1 Internal Medicine, Jawaharlal Nehru Medical College, Datta Meghe Institute of Higher Education and Research, Wardha, IND

**Keywords:** clinical outcomes, adjunctive therapy, intensive care unit (icu) stay, cholinergic crisis, magnesium sulfate, organophosphate poisoning

## Abstract

Organophosphate (OP) poisoning is a critical public health issue, particularly in agricultural regions where these compounds are extensively used as pesticides. The toxic effects of OP compounds arise from their inhibition of acetylcholinesterase, leading to an accumulation of acetylcholine and a subsequent cholinergic crisis, which can be fatal if not promptly treated. Traditional management of OP poisoning includes the administration of atropine and pralidoxime; however, these treatments often fall short of reducing the high morbidity and mortality associated with severe cases. Recent research has highlighted the potential of magnesium sulfate as an adjunctive treatment for OP poisoning. Magnesium sulfate exerts its beneficial effects through mechanisms such as calcium channel blockade and stabilization of neuromuscular junctions, which help mitigate the cholinergic hyperactivity induced by OP compounds. Clinical studies have shown that magnesium sulfate can significantly reduce the duration of intensive care unit (ICU) stays and improve overall patient outcomes. This narrative review aims to comprehensively analyze current insights into using magnesium sulfate to manage OP poisoning. It discusses the pathophysiology of OP poisoning, the pharmacological action of magnesium sulfate, and the clinical evidence supporting its use. Furthermore, the review will address the safety profile of magnesium sulfate and its potential role in current treatment guidelines. By synthesizing available evidence, this review seeks to establish magnesium sulfate as a game-changer in the management of OP poisoning, ultimately contributing to better clinical practices and patient outcomes.

## Introduction and background

Organophosphate (OP) poisoning poses a significant global health threat, especially prevalent in agricultural and industrial environments where these chemicals are extensively used. These compounds, present in pesticides and nerve agents, exert their toxicity by inhibiting acetylcholinesterase, which leads to the accumulation of acetylcholine at nerve endings and subsequent cholinergic crisis [1]. Rapid intervention is crucial due to the potentially life-threatening complications associated with OP poisoning. Current treatment protocols typically involve the administration of atropine to block muscarinic effects and pralidoxime to reactivate acetylcholinesterase, yet severe cases still carry substantial morbidity and mortality [2].

In recent years, magnesium sulfate has emerged as a promising adjunctive therapy in managing OP poisoning. Its mechanisms of action include calcium channel blockade and stabilization of the neuromuscular junction, countering the hyperactivity induced by OP compounds at cholinergic synapses [3]. This review critically examines the role of magnesium sulfate in critical care settings for OP poisoning management. This review aims to elucidate magnesium sulfate's therapeutic potential in reducing intensive care unit (ICU) stays and improving outcomes for patients affected by OP poisoning by synthesizing evidence from clinical studies and guidelines.

Magnesium sulfate's ability to mitigate the effects of OP poisoning lies in its modulation of calcium channels, which helps stabilize excitable membranes affected by excessive acetylcholine release [3]. This stabilization is crucial in preventing the cascade of events leading to respiratory failure and other severe complications in poisoned individuals. Moreover, its use as an adjunctive therapy alongside standard treatments like atropine and pralidoxime offers a multifaceted approach to managing OP poisoning, potentially enhancing overall patient recovery and reducing the duration of critical care interventions [4]. By evaluating the existing body of literature and clinical experiences, this review aims to comprehensively assess magnesium sulfate's efficacy and safety profile in OP poisoning. Insights gained from this analysis could inform future guidelines and protocols, highlighting magnesium sulfate as a valuable addition to the therapeutic arsenal against OP poisoning, ultimately aiming to improve patient outcomes and reduce the burden on critical care resources.

## Review

Mechanism of organophosphate poisoning

Pathophysiology of OP Poisoning

OP poisoning fundamentally disrupts normal neurological function by inhibiting acetylcholinesterase (AChE), a crucial enzyme responsible for breaking down acetylcholine (ACh) at nerve synapses and neuromuscular junctions. OP compounds bind to AChE and phosphorylate it, rendering the enzyme inactive. This biochemical blockade leads to an accumulation of ACh, the neurotransmitter responsible for transmitting signals across cholinergic synapses. The excess ACh then overstimulates both muscarinic and nicotinic cholinergic receptors throughout the body [4]. The resulting physiological effects manifest in a broad spectrum of symptoms. Muscular manifestations include weakness, paralysis, muscle cramps, fasciculations (involuntary muscle twitching), and tremors, reflecting the hyperactivity at neuromuscular junctions caused by ACh accumulation. Other systemic symptoms include increased secretions such as saliva and tears, gastrointestinal disturbances like diarrhea and vomiting, constricted pupils (miosis), excessive sweating, confusion, altered mental status, and cardiovascular effects such as hypertension. Severe cases may even lead to hypoglycemia due to dysregulated autonomic nervous system activity [4]. Beyond these acute effects, OP poisoning can also induce oxidative stress and neuroinflammation, contributing to long-term neurotoxicity. These processes underscore the potential for lasting neurological damage even after the acute phase of poisoning has been treated. Additionally, non-cholinergic mechanisms involving glutamate and adrenergic receptors may further exacerbate the overall toxicity of OP compounds [5]. While AChE inhibition and subsequent ACh accumulation represent the primary mechanism of OP toxicity, the involvement of secondary pathways highlights the intricate and multifaceted nature of OP poisoning. This complexity underscores the importance of comprehensive management strategies that address immediate symptoms and mitigate potential long-term neurological sequelae associated with OP exposure [6].

Clinical Manifestations and Severity Levels

**Figure 1:**
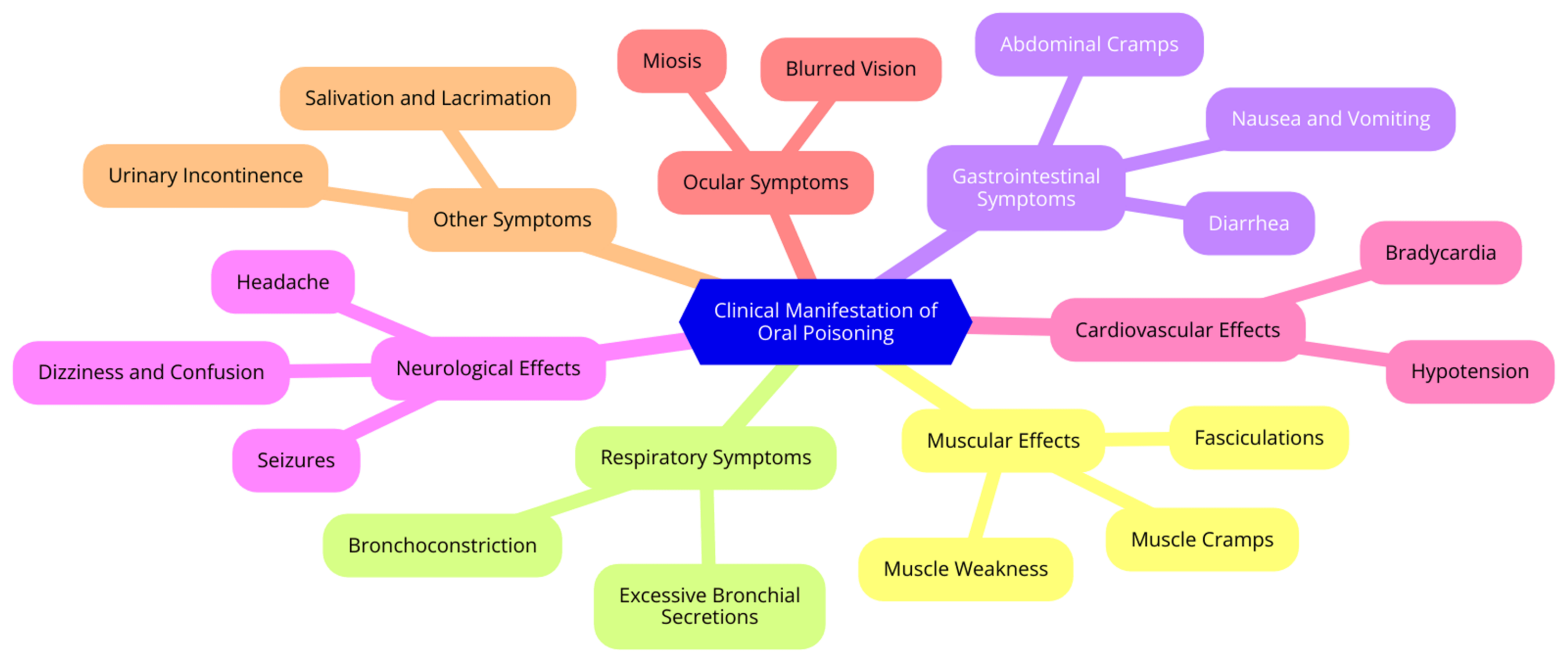
The clinical manifestations of OP poisoning Image credit: This image was created by one of the authors, Dr. Manikanta Nelakuditi. OP: Organophosphate.

OP poisoning can present with a wide array of clinical symptoms, the severity of which hinges on the dose and duration of exposure. Mild exposures may lead to minor effects such as narrowed pupils, impaired vision, runny nose, excess saliva, headache, nausea, and muscle weakness or twitching [6]. Moderate exposures escalate these symptoms, with patients experiencing dizziness, disorientation, difficulty breathing, excessive secretions, severe vomiting, diarrhea, and more pronounced muscle tremors and weakness, necessitating urgent medical attention to prevent deterioration [7]. The most severe and life-threatening cases of OP poisoning exhibit very narrowed pupils, confusion, agitation, convulsions, excessive body secretions, irregular heartbeat, respiratory depression, and possibly coma. Respiratory failure, particularly critical, can swiftly result in death without prompt intervention [8]. In addition to acute cholinergic effects, OP poisoning can trigger various long-term complications, including metabolic disorders, pancreatitis, neurological issues like muscle weakness and cognitive impairment, and fertility challenges. Early identification and proper management are crucial to mitigate short-term and long-term adverse outcomes [9]. Effective treatment involves prompt administration of antidotes such as atropine and oximes, alongside meticulous monitoring and supportive care in intensive care settings, particularly for patients with severe exposures and complications. Understanding the spectrum of clinical manifestations is essential for healthcare providers to ensure timely diagnosis and implementation of necessary interventions [3]. Figure 1 illustrates the clinical manifestations of OP poisoning.

Current Treatment Challenges and Outcomes

OP poisoning remains a significant global health challenge, especially prevalent in developing countries where it contributes to high mortality rates. Existing standard treatments, such as atropine, oximes, and supportive care, face several challenges. One major hurdle involves ethical concerns in clinically evaluating new emergency treatments, compounded by limited profitability in regions with high incidence rates of OP poisoning [10]. Despite these obstacles, ongoing research is exploring innovative approaches to enhance outcomes in OP poisoning. One promising adjunct therapy under investigation is magnesium sulfate (MgSO_4_). Studies suggest that MgSO_4_ may reduce ICU stays, decrease atropine requirements, and lessen the need for intubation in OP poisoning patients, although definitive evidence is still pending. Dosages ranging from 4 to 16 g have been studied, with 4 g generally well-tolerated [11]. In addition to MgSO_4_, other novel adjunct therapies are being explored. Gacyclidine, an antiglutamatergic compound, has shown benefits when combined with standard treatments like atropine, pralidoxime, and diazepam for nerve agent poisoning. Bioscavengers such as fresh frozen plasma or albumin are proposed to aid in clearing free organophosphates from the body. Hemofiltration and antioxidants are also under investigation as potential therapeutic options. Moreover, novel drug delivery methods, like nasal drops or sublingual tablets for rapid atropine administration, are being studied to improve treatment efficacy and accessibility [12]. Despite these promising advancements, significant challenges persist in developing and clinically validating these new approaches, particularly in the regions most affected by OP poisoning. Respiratory failure remains a common cause of death in severe cases, influenced by factors such as the type and extent of exposure and access to healthcare. Long-term exposure to OPs can lead to severe complications affecting multiple body systems [13]. While current standard treatments have limitations, ongoing research into novel adjunct therapies and delivery methods offers hope for improving outcomes in OP poisoning. Addressing these challenges requires sustained efforts to enhance treatment efficacy and accessibility, particularly in regions heavily burdened by this global health issue [14].

Role of magnesium sulfate

Mechanisms of Action in OP Poisoning

**Figure 2:**
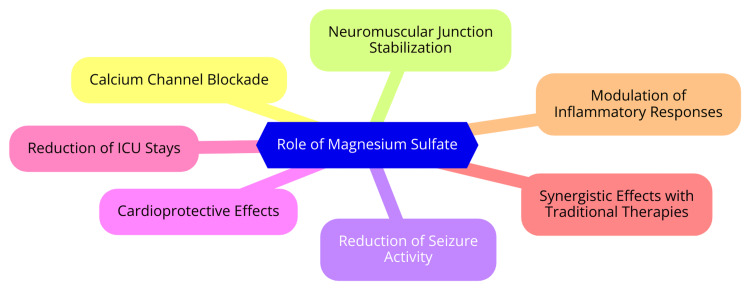
The role of magnesium sulfate in oral poisoning Image credit: This image was created by one of the authors, Dr. Manikanta Nelakuditi.

OP poisoning operates through multifaceted mechanisms, primarily centered on inhibiting the enzyme acetylcholinesterase (AChE). OP compounds bind to and deactivate AChE, accumulating acetylcholine (ACh) at cholinergic synapses in the peripheral and central nervous systems. This excess ACh triggers overstimulation of muscarinic and nicotinic receptors, characteristic of cholinergic syndrome symptoms such as excessive secretions, nausea, vomiting, and potentially fatal respiratory failure [15]. Beyond AChE inhibition, research indicates that OPs exert acute and long-term neurotoxic effects through non-cholinergic pathways. These include inducing status epilepticus, disrupting calcium regulation, generating oxidative stress, promoting neuroinflammation, and impairing synaptic function. The relative significance of these diverse pathways in OP toxicity remains an active area of investigation [4]. A critical consideration in OP poisoning is the reversibility of AChE inhibition. The extent of inhibition can range from reversible to irreversible, depending on whether "aging" occurs, which involves covalent bonding that renders the inhibition irreversible. Irreversible AChE inhibition poses greater challenges for treatment and may lead to persistent neurological impairments, underscoring the importance of early intervention and effective management strategies [16]. Individual susceptibility to OP toxicity is also influenced by genetic and environmental factors affecting the activity of paraoxonase 1 (PON1), an enzyme crucial in determining an organism's sensitivity to OP exposure. Understanding these complex mechanisms of OP toxicity is crucial for developing more targeted and effective treatments to improve patient outcomes [17]. Figure 2 illustrates the role of MgSO_4_ in oral poisoning.

Evidence Supporting Magnesium Sulfate Use

The role of MgSO_4_ as an adjunct therapy in the management of organophosphorus compound (OPC) poisoning has been extensively studied, yet the evidence regarding its effectiveness remains varied [18]. Some studies suggest the potential benefits of MgSO_4_ in this context. For instance, a double-blind, randomized controlled trial (RCT) indicated that a single infusion of 4 g of 20% MgSO_4_ upon admission to the ICU reduced the need for atropine, intubation, and ICU stay duration. However, it did not significantly affect mortality [11]. Moreover, a recent study comparing different MgSO_4_ doses (4, 8, 12, and 16 g) showed improved outcomes with higher doses, while 4 g was generally well-tolerated [19-21]. However, conflicting findings also exist. A prospective open-label trial found that intravenous MgSO_4_ at 4 g/day did not result in better outcomes compared to standard care alone, showing no significant differences in mortality, development of intermediate syndrome, need for mechanical ventilation, or hospital stay duration [20]. Another RCT reported statistically insignificant differences, although administering 4 g/day of MgSO_4_ for the initial 24 hours possibly reduced mortality, intermediate syndrome occurrence, and cardiovascular toxicity [22]. While some studies suggest the potential benefits of MgSO_4_ in reducing ICU stays and improving outcomes in OPC poisoning, the overall evidence remains inconclusive. This discrepancy underscores the necessity for larger, well-designed studies to clarify MgSO_4_'s efficacy and determine the optimal dosing regimen for managing OPC poisoning [18,19]. Continued research is essential to establish MgSO_4_'s definitive role as an adjunct therapy in this critical medical scenario.

Comparison With Other Treatment Options

The role of MgSO_4_ in managing OPC poisoning remains uncertain based on current research findings. Some studies suggest the potential benefits of MgSO_4_ as an adjunct therapy, while others do not significantly improve outcomes [20]. A double-blind RCT demonstrated that a single infusion of 4 g of 20% MgSO_4_ administered at ICU admission reduced the need for atropine, intubation, and length of ICU stay in OPC poisoning patients. However, this study did not find a significant difference in mortality between the MgSO_4_-treated and the control groups [20]. Conversely, a prospective open-label trial did not observe significant benefits of intravenous MgSO_4_ at a dose of 4 g/day compared to standard care alone. This trial reported no significant differences in mortality, development of intermediate syndrome, need for mechanical ventilation, or hospital stay duration between the two groups [23]. Another RCT suggested that administering 4 g/day of MgSO_4_ for the first 24 hours post-admission might decrease mortality, intermediate syndrome incidence, and cardiovascular toxicity in OPC poisoning patients. However, these differences were not statistically significant compared to untreated patients [24]. More recent research comparing varying doses of MgSO_4_ (4, 8, 12, and 16 g) indicated better outcomes with higher doses, while 4 g was generally well-tolerated. Despite these findings, the evidence regarding MgSO_4_'s efficacy as an adjunct therapy in OPC poisoning remains inconclusive [25]. While some studies indicate the potential benefits of MgSO_4_ in reducing ICU stays and improving outcomes in OPC poisoning, further well-designed research is essential to determine its role and establish the optimal dosing regimen conclusively. Continued investigation is necessary to guide clinical practice effectively in managing this critical condition.

Guidelines and recommendations

MgSO_4_ is widely utilized in various clinical scenarios, notably in managing preeclampsia and eclampsia. Current guidelines endorse MgSO_4_ as the primary treatment for preventing and controlling seizures in these conditions. The World Health Organization (WHO) recommends MgSO_4_ in concentrations of 500 mg/mL (50% w/v), available in 2-mL and 10-mL ampoules, facilitating its use in both the Pritchard (IV/IM) and Zuspan regimens [26]. In managing preeclampsia and eclampsia, a typical regimen involves administering an initial loading dose of 4-6 g intravenously over 15-20 minutes, followed by a maintenance infusion of 1-2 g per hour. Serum magnesium levels should be monitored every six to eight hours to maintain therapeutic levels between 2.0 and 3.5 mmol/L (4-7 meq/L). Careful monitoring for signs of toxicity is essential; these may include loss of patellar reflexes (at levels of 4-5 mmol/L), respiratory depression (5-7.5 mmol/L), and cardiac arrest (12.5-15 mmol/L) in severe cases [27]. Administration of MgSO_4_ should be conducted using an infusion pump with clear tubing labeling and Luer-lock connectors to prevent inadvertent disconnections or misadministration. An independent double-check by two nurses is recommended when initiating MgSO_4_, adjusting the dose, or during shift changes to ensure accuracy and patient safety. Immediate discontinuation of MgSO_4_ is advised if signs of toxicity develop, with calcium gluconate available for parenteral administration to alleviate symptoms. In severe cases or patients with renal impairment, hemodialysis may be necessary to facilitate magnesium elimination [28]. In addition to its established uses in obstetrics, MgSO_4_ is indicated for treating hypomagnesemia, managing cardiac arrhythmias associated with magnesium deficiency, and addressing constipation. Off-label applications include managing acute asthma exacerbations, treating torsades de pointes during advanced cardiac life support (ACLS), and preventing preterm labor. Adherence to these guidelines is crucial for optimizing patient outcomes and minimizing adverse events associated with MgSO_4_ therapy [29].

Areas needing further research

Further research into MgSO_4_ in the ICU setting is poised to refine dosing and administration protocols, particularly for specific patient groups. While current guidelines provide foundational recommendations, tailoring protocols for conditions such as OP poisoning or severe asthma exacerbations could significantly enhance therapeutic efficacy [30]. Investigating MgSO_4_'s mechanisms of action and therapeutic targets represents another critical avenue. Researchers can develop more precise treatment strategies by elucidating how MgSO_4_ reduces mortality rates and shortens ICU stays. Studies focusing on its molecular and cellular mechanisms and identifying biomarkers predictive of treatment response hold promise in optimizing therapeutic approaches [31]. Exploration into off-label uses of MgSO_4_, such as managing acute asthma exacerbations or preventing preterm labor, necessitates rigorous clinical trials within ICU settings. It is essential to establish its efficacy and safety for these applications through robust studies [32]. Furthermore, enhancing monitoring and toxicity management protocols is crucial. Developing novel biomarkers or refining existing monitoring methods can facilitate early detection and management of MgSO_4_ toxicity, thereby ensuring safer administration in critically ill patients [33]. Long-term outcomes research is equally imperative. While current investigations predominantly focus on short-term outcomes like mortality rates, comprehensively assessing MgSO_4_'s impact on patient quality of life, cognitive function, and overall health post-ICU discharge is essential for holistic patient care [34]. Addressing these research gaps promises to advance our understanding of MgSO_4_'s role in critical care, potentially leading to improved patient outcomes and guiding informed clinical decisions in ICU settings [30]. This multifaceted approach underscores the importance of ongoing research to refine MgSO_4_ therapies and optimize its benefits across diverse critical care scenarios.

## Conclusions

MgSO_4_ has emerged as a promising adjunctive therapy in the management of OP poisoning, offering mechanisms that counteract the cholinergic crisis induced by these toxic compounds. Evidence from clinical studies indicates that MgSO_4_ can significantly reduce the duration of ICU stays and improve overall patient outcomes. Despite traditional treatments with atropine and pralidoxime, the inclusion of MgSO_4_ provides an additional layer of efficacy, particularly in severe cases of OP poisoning. Its ability to stabilize neuromuscular junctions and block calcium channels addresses critical aspects of the pathophysiology of OP poisoning, making it a valuable addition to current therapeutic protocols. Further research and clinical trials are needed to solidify guidelines and optimize dosing strategies, but the potential of MgSO_4_ to enhance patient recovery and reduce healthcare burdens is undeniable. This review underscores the importance of integrating MgSO_4_ into standard treatment regimens for OP poisoning, ultimately aiming to improve survival rates and quality of care for affected individuals.
